# Risk behaviors among migrant adolescents in Italy

**DOI:** 10.1093/eurpub/ckac130.081

**Published:** 2022-10-25

**Authors:** E Koumantakis, M Bersia, P Berchialla, L Charrier, RI Comoretto, A Borraccino, P Lemma, P Nardone, A Vieno, P Dalmasso

**Affiliations:** 1 Department of Public Health and Pediatrics, University of Torino, Turin, Italy; 2 Post Graduate School of Medical Statistics, University of Torino, Turin, Italy; 3 Department of Clinical and Biological Science, University of Torino, Orbassano, Italy; 4 CNaPPS, Italian National Institute of Health, Rome, Italy; 5 Department of Developmental Social Psychology, University of Padova, Padua, Italy

## Abstract

**Background:**

Over the last decade, the student population migrated to Italy has quadrupled, but studies on their potentially harmful behaviors such as substance use are still scarce. The aim of this research is to monitor risk behaviors among migrant adolescents in Italy and provide appropriate indications for the definition of targeted policies and interventions.

**Methods:**

A representative sample of 15 year-old adolescents was drawn from the 2018 Italian Health Behavior in School-aged Children (HBSC) survey data. Smoking habits, alcohol consumption and drunkenness were investigated and differences with Italian peers were assessed.

**Findings:**

Results were based on more than 18,500 students, of which 16% were migrants: 32.7% from Western countries (We), 32.5% from Eastern European countries (Ee), and 34.8% from non-Western/non-European countries (nW). Compared with natives, students from nW countries showed a lower risk of smoking habits (OR: 0.72, 95%CI: 0.58-0.89) and weekly alcohol consumption (OR:0.57, 95%CI:0.43-0.75), whereas drunkenness was more prevalent among Ee migrants (OR: 1.42, 95%CI: 1.10-1.83). Overall, both migrant and Italian girls showed a lower risk of unhealthy behaviors than boys.

**Conclusions:**

Compared to the native counterparts, migrant adolescents showed differences in substance use according to their ethnic background. We observed two different immigration patterns: the Western immigrants, who came from countries with higher affluence and share similar risk behaviors with native peers, and non-Western immigrants, who came from less affluent countries and seemed to maintain the risk behaviors of their culture of origin.

**Key messages:**

- More in-depth studies that include specific information on migrant culture are needed to explain the observed differences in risk behaviors among young migrants.
- Our findings suggest that interventions aiming at smoking and/or drinking reduction should focus mainly on the male migrant adolescents.

